# Methods of Incorporating Plant-Derived Bioactive Compounds into Films Made with Agro-Based Polymers for Application as Food Packaging: A Brief Review

**DOI:** 10.3390/polym12112518

**Published:** 2020-10-28

**Authors:** Gislaine Ferreira Nogueira, Rafael Augustus de Oliveira, José Ignacio Velasco, Farayde Matta Fakhouri

**Affiliations:** 1 Academic unit of Passos, Minas Gerais State University, Passos 37900-106, MG, Brazil; gislainefnogueira@gmail.com; 2 School of Agricultural Engineering, University of Campinas, Campinas 13083-875, SP, Brazil; augustus@feagri.unicamp.br; 3 Department of Materials Science and Engineering, Universitat Politècnica de Catalunya, Carrer Colom 114, E-08222 Terrassa, Spain; jose.ignacio.velasco@upc.edu; 4 Faculty of Engineering, Federal University of Grande Dourados, Dourados 79804-970, MS, Brazil

**Keywords:** food packaging, antioxidant activity, antimicrobials, natural compounds, biopolymers, agricultural products

## Abstract

Plastic, usually derived from non-renewable sources, is among the most used materials in food packaging. Despite its barrier properties, plastic packaging has a recycling rate below the ideal and its accumulation in the environment leads to environmental issues. One of the solutions approached to minimize this impact is the development of food packaging materials made from polymers from renewable sources that, in addition to being biodegradable, can also be edible. Different biopolymers from agricultural renewable sources such as gelatin, whey protein, starch, chitosan, alginate and pectin, among other, have been analyzed for the development of biodegradable films. Moreover, these films can serve as vehicles for transporting bioactive compounds, extending their applicability as bioactive, edible, compostable and biodegradable films. Biopolymer films incorporated with plant-derived bioactive compounds have become an interesting area of research. The interaction between environment-friendly biopolymers and bioactive compounds improves functionality. In addition to interfering with thermal, mechanical and barrier properties of films, depending on the properties of the bioactive compounds, new characteristics are attributed to films, such as antimicrobial and antioxidant properties, color and innovative flavors. This review compiles information on agro-based biopolymers and plant-derived bioactive compounds used in the production of bioactive films. Particular emphasis has been given to the methods used for incorporating bioactive compounds from plant-derived into films and their influence on the functional properties of biopolymer films. Some limitations to be overcome for future advances are also briefly summarized. This review will benefit future prospects for exploring innovative methods of incorporating plant-derived bioactive compounds into films made from agricultural polymers.

## 1. Introduction

Most food packaging is produced from synthetic materials from non-renewable sources, which, despite having excellent barrier and resistance properties, are causing serious environmental problems due to the generation of high amounts of non-degradable solid waste [1]. However, the use of packaging is essential. Apart from its basic function of containing the food, it also plays a fundamental role in controlling interactions between food and the environment, protecting and helping to maintain product quality [2,3].

This factor, along with environmental concerns, in combination with consumer demands for high quality eco-friendly products that are related to those found in nature (natural products) have driven the development of new technologies, such as the production of biodegradable films from polymers from renewable sources [1,2,4], for the development of packaging materials.

The biodegradability of plastic materials is defined as their ability to decompose through the enzymatic action of microorganisms [5]. The polymer degradation in a bioactive environment occurs by material fragmentation and subsequent mineralization. The action of heat and moisture as well as enzymatic activity of microorganisms abbreviate and fade the polymer chains, resulting in fragmentized residues of the polymer. These polymer fragments can only be considered biodegradable if they are consumed by microorganisms as food and energy source converted at the end of the degradation process into carbon dioxide (CO_2_), water (H_2_O) and biomass under aerobic conditions and hydrocarbons, methane and biomass under anaerobic conditions [6].

Polysaccharides, such as starch [7,8,9], cellulose [1,2] alginate sodium [3,10], pectin [4,11], chitosan [8,12], gums [13,14,15]; and proteins, such as whey [10,16], soy [17,18], gluten [19,20,21] and gelatine [10,19,22,23] are among the most employed biopolymers in the development of biodegradable films. These natural biopolymers are commonly used due to their abundance in nature, biodegradability and edibility. Among these natural biopolymers, starch stands out for its easy processing and low cost [1,7,8,24]. Among the techniques used for the production of these films, there are casting, pressing and extrusion followed by blowing [23].

In addition to biopolymers, plant-derived bioactive compounds such as essential oils, vitamins, minerals, polyphenols, carotenoids, among others, are widely distributed in nature. Different parts of plants, such as leaves, flowers, seeds and roots can potentially be used in the production of environment-friendly films with functional properties due to their biological nature [25]. Some bioactive compounds have antioxidant [26,27] and antimicrobial activity [3,27,28].

The combination of biopolymers with natural bioactive compounds has enabled the development of bioactive films with new and/or improved properties, i.e., antioxidant [26,27] and antimicrobial [3,27,28] effect, innovative colors [11,29], and customized barrier and mechanical properties [11,29,30].

The incorporation of bioactive compounds into biodegradable films has been extensively studied [3,9,28,29,31,32]. The use of inherently bioactive biopolymer-based materials [33,34,35] as well as the incorporation, direct or by sprinkling, of free or encapsulated bioactive compounds into the film-forming solutions [26,29] are some of the techniques employed for their production.

The application of bioactive films in packaging as a strategy for extending the shelf life, stability and safety of food products has shown great potential [36,37,38]. In the food industry, microbial deterioration and lipid oxidation are major problems to be overcome in order to increase the shelf life of food products [25]. The release of bioactive compounds from films into foods prevents the oxidation of lipid compounds present in their composition [37], as well as the growth of microorganisms [38], improving their shelf life. Thus, the use of natural bioactive compounds in biodegradable films has proved to be a potential alternative for solving this problem in the food industry and for replacing traditional packaging [25]. Previous review papers have summarized the most recent trends on the strategies used for stabilizing bioactive compounds for inclusion in packaging materials [39] and for release control of active compounds from food active packaging systems [40]. Given the relevance and scope of the topic and the large amount of scientific research addressing the incorporation of bioactive compounds in films, more review articles are still needed. According to all the facts, this article provides an overview of the types of agro-based polymers from renewable sources and plant-derived bioactive compounds used, as well as the latest trends on methods used for their inclusion in biodegradable films. In addition, an in-depth description of each method of producing bioactive films and their functional properties is presented.

## 2. Main Agro-Based Polymers from Renewable Sources Used in the Development of Biodegradable Film

**Figure 1:**
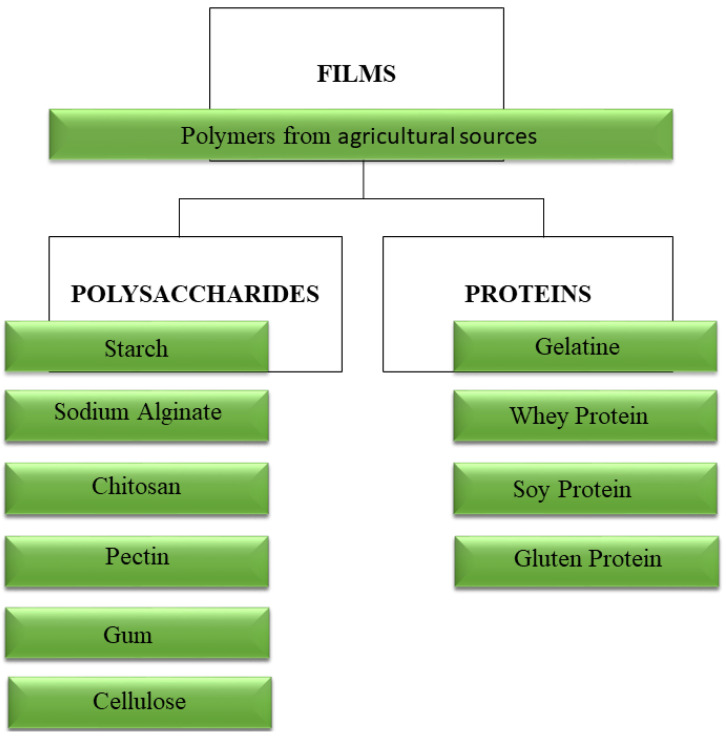
Main polymers of agricultural origin from renewable sources used in the development of biodegradable films.

Environmental concerns regarding the disposal of non-biodegradable materials, and in accordance with the circular economy values, include: use of renewable sources, preservation of fossil raw materials, landfill waste reduction and carbon dioxide (CO_2_) emission reduction. Several studies involving biodegradable polymers of agricultural origin (Figure 1) with lipids, plant-based proteins (zein, soy, pea, gluten), animal-based proteins (gelatin, whey, casein), and polysaccharides (starch, chitosan, sodium alginate, pectin, gums (and ligno-cellulosic (straws and wood)) are emerging.

Some characteristics and properties of these biopolymers of agricultural origin have been reported. The most abundant organic compound in nature is cellulose, its derivatives with the methylcellulose, hydroxypropylmethylcellulose and carboxymethylcellulose are widely used as raw materials that form edible films, as they are naturally odorless and tasteless [41]. However, cellulose derivative films showed poor properties as a barrier to water vapor due to their hydrophilicity [42]. Likewise, films produced with Carrageenan gum exhibited limitations regarding their water vapor permeability and water resistance [43]. Gums are another group of polysaccharides that have been used as film forming agents and are produced naturally by some botanical (trees and shrubs, seeds and tubers), algae (red and brown algae) or microbial sources [44].

Starch, a polysaccharide composed of two macromolecules, amylose and amylopectin, is used in its native and modified form. In addition, due to different proportions of amylose and amylopectin present in different starch sources, unconventional starch sources have been increasingly used in the production of edible films, such as arrowroot starch [9] and black rice starch [45]. The two components can be separated, allowing new blends with other proportions and, thus, increasing their use [46]. Starch has high amylose content, a desirable feature for the production of films with good technological properties and stronger and more flexible mechanical characteristics [47]. Corn, potato, rice, wheat and cassava starches are the most commercially used [48].

Alginate and pectin are anionic polysaccharides. Pectin is derived from the plant cell wall and it is obtained commercially by aqueous extraction of pectic material from some fruits, being found in higher amounts in citrus fruits and apples [49]. It consists mainly of the methoxy esterified α, d-1, 4-galacturonic acid units [50]. Alginate is composed of β-d-manuronic acid and α-l-guluronic acid, joined linearly by α-1,4 glycosidic bonds, obtained from cell walls of brown algae [51]. Both biopolymers are widely applied in the food industry for being able to form gels with the presence of divalent cations, such as calcium ions. Moreover, they are able to create biodegradable edible films with measurable characteristics [52]. The combination of both polysaccharides generated continuous, homogenous and transparent films [53]. The addition of alginate into pectin-based formulations improved the strength of zinc ions crosslinking network [54]. Chitosan (poly (1,4-β-d-glucopyranosamine)), is a biodegradable cationic polymer derived from chitin, a polysaccharide of animal origin. Chitosan shows antimicrobial properties [55] and the ability to form films [56]. According to Vargas et al. [56], due to its high to water vapor permeability, its barrier properties can be improved by its combination with other hydrocolloids. Chitosan is an ideal biopolymer for the development of antimicrobial films due to its non-toxicity [57], biocompatibility [58], biodegradability and intrinsic antimicrobial action.

The most widely used proteins in the production of edible films are gelatin [19,23], whey protein isolate [59], soy [17,18] and gluten [19,20,21]. Gelatine is classified and commercialized according to its strength, or “bloom” [60]. “Bloom” and viscosity are the main rheological characteristics of gelatine and are usually the result of the manufacturing process applied. The viscoelastic properties are related to the amino acid composition, average molecular weight and the degree of polymerization of the chain [61]. There are reports of the use of gelatine type A [23] and type B [62]. Gelatine forms films with high transparency and good tensile strength; in order to improve its mechanical properties, blends with different hydrocoids are widely used [19,63].

Bovine milk whey is a by-product of the dairy industry. It represents the aqueous portion of milk that separates from the clot during cheese making or casein production and consists of a complex mixture of globular proteins (~0.6%), lipids, minerals and lactose, in water (93%) [64]. Drying and removing non-protein components from whey leads to commercial products such as concentrates (whey protein concentrate—WPC, with 25 to 80% proteins) or isolates (whey protein isolate—WPI, with proteins concentration ≥90%) of whey proteins, which are widely used in the food sector due to their functional properties as gelling agents, emulsifiers and foam stabilizers [65].

Whey protein isolate (WPI) has the ability to form films with a wide range of functional properties depending on their structural cohesion [59]. Native WPI films presented rapid water dissolution, showing favorable edible characteristics [59]. Films produced with heat-denatured WPI (HWPI) proved to be water-insoluble with good mechanical properties and excellent oxygen, aroma and oil barrier properties at relative low humidity [66]. Wheat gluten is a general term for water-insoluble proteins separated from wheat flour. It consists of a mixture of peptide molecules considered globular proteins. The cohesiveness and elasticity of gluten produce integrity and facilitate the formation of films [67]. Gluten can be obtained by pressing an aqueous mass of wheat flour and gently washing the starch and other soluble materials in a dilute acid solution or in an excess of water. It can be separated into two fractions: (i) gliadin, soluble in 70% ethanol, and (ii) glutenin, insoluble in ethanol [68]. These authors made films based on wheat gluten and obtained high elongation values. Gontard et al. [69] studied the addition of various concentrations of lipids into edible gluten films developed by the casting technique as barrier components to water vapor permeability. The effects of this addition depended on the characteristics of the lipids and their interactions with the protein structural matrix.

Soy protein isolate (SPI) is a byproduct of the soybean oil industry, it comprises a set of macromolecules of varied sizes and structures formed from 18 different amino acid residues, being constituted by the soy storage proteins [70]. Due to its availability, environmentally friendly nature and excellent film-forming property, soy protein isolate has been widely accepted for exploitation in protein-based films [71]. SPI-based film usually shows lower permeability oxygen (O_2_) behavior in comparison to films based on low-density polyethylene, methylcellulose, starch and pectin. According to a study by Giacomelli [70] on thermal and morphological properties of films made with soy protein isolate, the thermal degradation of these films occurs in a single step, starting at 292 °C. However, due to their high hydrophilicity, films do not present satisfactory mechanical properties or a water vapor barrier for applications such as packaging [72].

Despite the promising characteristics of edibility, biocompatibility and biodegradability presented by polymer from agricultural sources, it is still evident that there are limitations to be overcome for the commercial success of these films (mainly low elongation, low resistance to gases and liquids), especially when compared to synthetic plastics [73]. In general, it is noted that films produced from polysaccharides tend to have a good barrier against gases, but have low resistance to water vapor and low mechanical resistance, while the films produced from proteins also have low resistance to water vapor, but good mechanical resistance [73].

In addition, most films made from polymers of agricultural origin are obtained on a laboratory scale, using the casting method. This method is based on the dispersion of macromolecules into a suitable solvent or mixture of solvents, thus obtaining the film-forming suspension that is subjected to thermal gelatinization. The resulting solution is deposited in molds of relatively small sizes (90 mm × 15 mm [11]; 14 cm × 14 cm [74]; 12 cm diameter [26]; 14.3 cm diameter [30]) for drying and solvent removal. In order for the properties of the resulting films not to be negatively affected, low temperatures (around 25 °C to 45 °C [3,11,74,75]) are required in the drying step, leading to long drying times (2–3 days). The difficulty of making films of large size (>25 cm), with precise thickness (local variations in thickness) and short drying times, make current methods of manufacturing laboratory-scale films unsuitable for expansion into industrial production [73].

## 3. Most Common Plant-Derived Bioactive Compounds Incorporated into Biodegradable Films for Development of Bioactive Films

In recent years, bioactive compounds have attracted the attention of scientists, researchers and the world’s population, as their consumption has been associated with beneficial effects on physical and mental health because they provide crucial biological effects in the prevention and treatment against a wide range of diseases. Some bioactive compounds have antimicrobial [2,12,25], antioxidant [12,37,75], anticancer, anti-inflammatory and/or anti-neurodegenerative activities [76].

Widely distributed in nature, bioactive compounds are mainly secondary metabolites of plants, presenting both nutritional value and other functions in their metabolism, such as a growth stimulator and a protector against biotic and abiotic stress [77].

**Figure 2:**
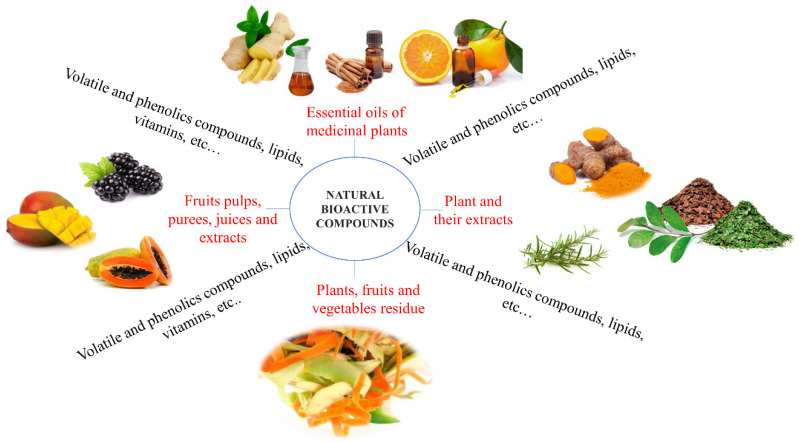
Examples of plant-derived bioactive compounds.

Leaves, flowers [3], fruits, vegetables [11], seeds, grains [31,78], rhizomes and roots [32], of various types of plants are good sources of diversified bioactive compounds including phenols, essential oils, proteins, terpenoids and flavonoids, among others (Figure 2), as listed:

- plants and their extracts as a source of phenolic compounds: of *Plantago lanceolata, Arnica montana, Tagetes patula, Symphytum officinale, Calendula officinalis* and *Geum urbanum* [79]; turmeric [32]; *Acca sellowian* [80]; Chinese chive root [27]; tea polyphenol [28]; rosemary [81]; yerba mate [82]; jujube leaf [83];
- essential oils from medicinal plants as a source of volatile and phenolics compounds and lipids: *M. pulegium* L., *A. Herba alba Asso, O. basilicum* L. and *R. officinalis* L. [3]; green coffee beans (*Coffea arabica* L. [31]); thyme essential oil [84]; *Ziziphora clinopodioides* essential oil [85]; orange essential oil [86]; cinnamon leaf essential oil [13]; black pepper essential oil and ginger essential oil [87]; rosemary essential oil [88]; *Satureja Khuzestanica* essential oil [89];
- fruit pulps, purees, juices and extracts as a source of phenolic compounds and vitamins: guabiroba [74]; blackberry [26], pomegranate [90]; açai [91]; papaya [92], blueberry [93]; mango; acerola; seriguela [94]; anthocyanins from jambolan fruit (*Syzygium cumini*) [95]; mulberry anthocyanin extract [96]; papaya puree [97]; mango and acerola pulps [98]; acerola [99].
- plants, fruits and vegetables residue flour or extract: sweet orange (*Citrus sinensis*), passion fruit (*Passiflora edulis*) and watermelon (*Citrullus lanatus*), whereas the vegetables were zucchini (*Cucurbita pepo*), lettuce (*Lactuca sativa*), carrot (*Daucus carota*), spinach (*Spinacea oleracea*), mint (*Menthas* p.), yams (*Colocasia esculenta*), cucumber (*Cucumis sativus*) and arugula (*Eruca sativa*) [11]; pomelo peel flours [28], *Acca sellowiana* waste by-product (feijoa peel flour, [80]); roasted peanut skin extract [100]; pine nut shell, peanut shell [83]; ethanolic red grape seed extract [85].

Plant-derived bioactive compounds are being considered interesting ingredients for the production of biodegradable and bioactive films due to their natural origin and functionality [25]. Studies have shown that the incorporation of plant extracts and fruit pulps as sources of bioactive compounds or isolated bioactive compounds into film-forming solutions cause antioxidant and antimicrobial effects on the resulting films, extending their application in bioactive and biodegradable films or packaging [2,3,12,27,36,101]. Recently, Benbettaïeb et al. [102] carried out an in-depth review on the mechanisms involved in the antimicrobial and antioxidant activity of edible bioactive films for food applications.

There is a wide and growing list of bioactive compounds that have been or are being incorporated into films, and phenolic compounds (polyphenols, phenolic acids, coumarins, volatiles phenols and so on) are the most common ones. The plant-derived bioactive compounds are incorporated also to contribute to the general quality [56,63,68,93,103], safety [25,86,92,104], nutritional value [9], organoleptic characteristics (color, smell and taste) [9,26,95], convenience and preservation of foods [105]. Some plant-derived bioactive compounds, such as some polyphenols present in apples [106], in teas [28], extract of germinated fenugreek seeds [107], *R. officinalis*, *A. herba alba Asso*, *O. basilicum* L., *M. pulegium* L. [3], *Acca sellowiana* waste by-product (feijoa peel flour) [80], for example, can perform multiple functions and can be used as antioxidants and antimicrobial agents simultaneously.

There is still a wide range of plant-derived bioactive compounds that have not been characterized in terms of physical–chemical properties, toxicity and edibility. This fact implies that extensive investigations, including tests that assess the in vitro and in vivo (food product) efficiency of plant-derived bioactive compounds, are still needed for improving food safety. The choice of specific plant-derived bioactive compounds for application depends on their bioactive properties (antioxidant, antimicrobial), availability and cost–benefit ratio [105].

One of the great challenges to be overcome for future advances and insertion of bioactive and biopolymer films into the market is the standardization of the functional, barrier and mechanical properties presented by them. It is known that the amount of biopolymers (starch, protein, fibers, cellulose), as well as bioactive compounds (carotenoids, phenolic compounds, vitamins, among others) present in agricultural products and their byproducts (fruits, vegetables, grains, seeds, rhizomes, roots) vary in composition and quantity, depending on the conditions adopted in agricultural practices: planting (soil, irrigation, location); harvest (maturity degree); post-harvest (processing and storage conditions); part of the plant; and, mainly for bioactive compounds, how much the plant responds to environmental aggressions [11,35,108,109].

A recent study showed the influence of different factors (four parameters of soil physical properties, twelve parameters of soil chemical properties, thirty-year climate records and genetic diversity) on the bioactive compounds of *Glechoma longituba*, a plant widely spread in China that has long been used as a beneficial health ingredient in human diet. In addition to the great variations in the levels of extracts soluble in ethanol, total flavonoids, chlorogenic acid, caffeic acid, rosmarinic acid, oleanolic acid and ursolic acid proven among different populations of *Glechoma longituba*, it was observed that the chemical property of the soil and the climatic condition exerted significant influences on the concentrations of bioactive compounds present in the plant [108]. More studies are necessary to provide information on the ideal planting conditions to modulate the bioactive properties of the plants. Variations in the types and amounts of bioactive compounds generate films with diverse properties.

One of the ways to decrease the variability of bioactive compounds is to extract or purify them. Recent reviews have reported the use of compressed fluids, mainly under sub- and supercritical conditions, for the extraction of bioactive components from natural matrices [110,111]. However, the extraction or purification of the compounds generates biopolymer materials and bioactive compounds with low variability and higher costs compared to their natural sources [77,111].

Moreover, when the bioactive compound is extracted and purified, it is more exposed to external adverse conditions (light, pH, oxygen, etc.), which facilitates its volatilization and oxidation during the production of films due to processing conditions (temperature, pH) [30,35,82], which also contributes to this difficulty in standardizing the properties of bioactive films.

## 4. Methods for Incorporating Bioactive Compounds

Currently, several studies have investigated the incorporation of bioactive compounds into biopolymer-based films for application such as active food packaging [3,12,25,26,27,35,36,74,78,80,94]. Unlike conventional packaging, active packaging (AP) has some characteristics that allow the packaging material to interact positively with food and the environment, extending product shelf life. The active packaging can come in various forms, such as sachets or compresses containing bioactive compounds, coating or adsorption of active compounds on the polymer surface; immobilization of active compounds in polymers by ion or covalent bonds; polymeric films with direct incorporation of bioactive substances or packaging even produced with inherently bioactive polymers [39,40].

**Figure 3:**
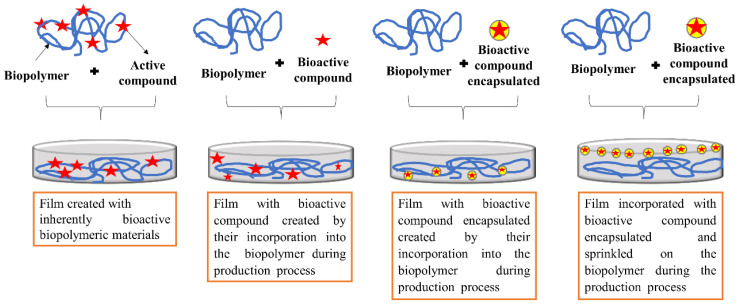
Schematic preparation of bioactive films agro-based polymers incorporated with plant-derived bioactive compounds.

The incorporation of bioactive compounds into films must be in accordance with the characteristics of the desired packaging system, the bioactive compound used, as well as the food to which these films will be applied. As a result, various ways of incorporating plant-derived bioactive compounds into film-forming solutions produced by agro-based polymers have been reported, and the most commonly used are:

(a) producing the film using inherently bioactive biopolymeric materials from agricultural products and their byproducts [34,35,112,113];

(b) directly mixing bioactive compounds with biopolymers during the film producing process [3,26,31,32,74,75,79];

(c) encapsulating the bioactive compounds, keeping them trapped inside a wall material, forming micro or nanoparticles. Then, performing the incorporation by physical and direct mixing of micro or nanoparticles with the biopolymer in the film producing process [30,36,114,115,116];

(d) encapsulating the bioactive compounds, keeping them trapped inside a wall material, forming micro or nanoparticles. Then, carrying out the incorporation by spraying micro or nanoparticles onto the biopolymer during in the film producing process [29,117,118]. A graphic illustration of the preparation of films with bioactive compounds is shown in Figure 3. It is important that studies test the compatibility and concentration of the plant-derived bioactive compound in relation to the film-forming polymer because the properties of the resulting film depend on their combination and the proportion between them, as demonstrated by Silva-Weiss [105]. The chemical interactions between bioactive compounds and biopolymers depend on their chemical and structural characteristics (stereochemistry, conformational flexibility and molecular weight), concentration and pH, which in return affect the structure as well as the functionality of the resulting films. Particularly, depending on the characteristics of the bioactive compound, the concentration to be added to the film-forming solution must be evaluated. In general, very high concentrations in relation to the polymer mass are not widely used because they can generate undesirable odors, turbidity and / or precipitation [105,119].

### 4.1. Films Made with Inherently Bioactive Biopolymer-Based Materials

In recent years, researches have focused on the sustainable use of agricultural and agro-industrial products and byproducts, which include bagasse, barks, stalks, seeds, among others, as raw materials for the production of bran, flour, fibers, starch extraction [11,33,34,35,120,121] or bioactive compounds [122]. These types of materials have great potential because, in addition to containing biopolymers such as proteins, fibers and starch, they may also contain bioactive compounds such as phenolic compounds with antioxidant and antimicrobial activities in their composition, which allow the production of bioactive films with functional properties differentiated from conventional materials [34,35].

Films produced with saffron flour obtained from the extraction residue of saffron dye showed antioxidant activity due to the presence of curcuminoids in the flour [34]. Light blue films were produced using blue cornflour as a base. For the authors, the blue color displayed by the films confirms that the conditions applied in their production did not affect their antioxidant capacity [112]. Bombacaceae gum (BG), a low-cost exudate gum, made it possible to develop yellowish-orange films. According to the authors, the film exhibited antioxidant activity with 1,1-diphenyl-2-picrylhydrazyl radical (DPPH) and 2,2′-azino-bis-3-ethylbenzothiazoline-6-sulphonic acid (ABTS) radical scavenging activities of 52.63% and 54.74%, respectively, due to the phenolic compounds inherent in the gum [13]. Films with high levels of compounds with antioxidant properties were produced with red rice flour and red rice starch, which slowed down the oxidation process of sunflower oil, displaying a protective effect [113].

Maniglia et al. [35] used babassu mesocarp, a byproduct of the babassu oil extraction industry, to produce bioactive films. These authors developed films with babassu mesocarp, or with starch isolated from babassu mesocarp, by steeping in water, alkaline or acid medium. Both materials used in the production of the films and the films obtained from them showed phenolic compounds and antioxidant activity. The different methods used for obtaining starch also influenced the different levels of amylose, lipids, fibers and total phenolic compounds, which reflected in the mechanical and functional properties of the films obtained by these methods.

The production of bioactive films with inherently bioactive biopolymer-based materials, i.e., those that naturally contain bioactive compounds in their composition, presents the following advantages:

- elimination of the need for incorporating materials that are sources of bioactive compounds or isolated bioactive compounds during their preparation, which can lead to a reduction in production costs;
- development of films with special functional properties, such as antioxidants and antimicrobials, and with added value, since they carry bioactive compounds capable of delaying the discoloration and oxidation of other compounds, preventing bacterial growth, as well as prolonging the shelf life of food products [11].
- Utilization of agricultural and agro-industrial products and byproducts as raw material can contribute to minimizing environmental issues caused by their disposal.

Therefore, it is necessary to explore the potential of biopolymer-based materials obtained mainly from agricultural products and byproducts, derived from the processing of fruits and vegetables, which are rich sources of low-cost bioactive biomaterials [11].

### 4.2. Films Incorporated with Bioactive Compounds Directly into Agro-Based Polymers

Instead of using inherently bioactive products, bioactive films can also be developed by incorporating natural raw materials that are sources of bioactive compounds or isolated bioactive compounds into the biopolymer film-forming solution during preparation. Some studies incorporate the bioactive compounds into the biopolymer during solubilization [31,32], while others previously solubilize the biopolymer and then incorporate the bioactive compounds to produce the film-forming solution [3,9,74,75,79,117]. The different stages of the incorporation of bioactive compounds into film-forming solutions result from their solubilization characteristics, the amount of material to be added, their water content and instability in production conditions (pH, temperature, among others). This is the most common type of incorporation of bioactive compounds in films. This technique allows a homogeneous distribution of the active compound in the polymeric matrix and a slow release to the surrounding environment [40].

The films generated different functional properties when different materials, sources of bioactive compounds or isolated bioactive compounds, were incorporated into the film-forming solution. The films incorporated with tea polyphenol [28], Chinese chive root extract [27], essential oils of *R. officinalis* L., *A. herba alba Asso*, *O. basilicum* L. and *M. pulegium* L. [3] and *Acca sellowiana* waste byproduct (feijoa peel flour [80]) exerted both antioxidant and antimicrobial activity. The incorporation of green coffee oil and gamma-aminobutyric acid residues affected the color and provided the films with a high antioxidant capacity based on carboxymethyl cellulose [31] and pectin [123], respectively; adding curcumin promoted antioxidant properties just as well as in whey protein isolate films, in addition to intensifying their coloration with yellow to red tones [75].

Brito et al. [11] produced yellow, malleable, homogeneous and water-soluble films with fruit and vegetable residual flour (including peels, pits, seeds and stalks) with different granulometries and pectin levels. The residual flour with lower granulometry (<150 μm) had higher protein content. This flour, when enriched with pectin (0.25%) significantly reduced the hygroscopicity of the film, in addition to producing better mechanical properties. In contrast, the flour with higher granulometry (425–500 μm) was rich in dietary fibers, showing potential for application as a reinforcement material (micro/nanometric fibers) for biodegradable films. For the authors, flours produced with residues and with different granulometries can have different applications, depending on the size of the particles and composition.

### 4.3. Films with Encapsulated Plant-Derived Bioactive Compounds Developed by Their Incorporation Directly or by Spraying into the Biopolymer during Production Process

Some natural bioactive compounds show instability when exposed to high temperatures, light, pH, oxygen, among other processing conditions [124], which can alter their functions and properties if incorporated directly into the film-forming solution.

The encapsulation of bioactive compounds is one of the ways to stabilize and maintain their viability and extend their shelf life. Encapsulation refers to the process of trapping the bioactive compound into microsystems (microparticles) or nano-systems (nanoparticles) of structural engineering in order to develop a thermodynamic and physical barrier that protects against adverse conditions (heat, humidity, oxidation, chemical reactions or other extreme conditions), releasing it when necessary [125].

The strategy of incorporating encapsulated bioactive compounds into the film has been used for: (i) maintaining the stability and viability of active compounds when exposed to unfavorable conditions; (ii) improving compatibility between packaging polymer and bioactive compound (increased miscibility of lipophilic compounds with hydrophilic biopolymeric materials); (iii) increasing the availability of the bioactive compound; (iv) controlling the release of these compounds by specific stimuli [39].

The encapsulation of some bioactive compounds enables their use in packaging applications, which is the case of 2-phenyl ethanol, for example. The encapsulation of 2-phenyl ethanol was essential to reduce losses due to volatilization or degradation during the film production process. Zarandona et al. [30] observed this behavior when they studied the release of 2-phenyl ethanol from chitosan films with free 2-phenyl ethanol and chitosan films with β-cyclodextrin: 2-phenyl ethanol (encapsulated) during immersion of the films in 95% ethanol for 4 days. The results of releasing the films without the inclusion complex indicated that 2-phenyl ethanol was lost before or during the preparation of the films due to its high volatility, and only 8% of the total bioactive was retained in the film, while more than 90% of the 2-phenyl ethanol was retained in the films with the inclusion complex, confirming the improvement on the retention of the bioactive with its encapsulation [30]. Different methods have been used for encapsulating bioactive compounds so that they can later be incorporated into a film-forming solution. Thymol nanoemulsions, an antimicrobial essential oil, was produced by mixing gelatin and soy lecithin [115]. The lyophilization method was applied by Rodsamran et al. [126] for the microencapsulation of phenolic compounds from fresh rice extract, and from blackberry pulp by Nogueira et al. [29]. The spray drying technique was also used for the microencapsulation of blackberry pulp [29]. Paglione et al. [127] observed the effect of oregano essential oil microencapsulated by ionic gelation on soy protein concentrate. The internal ionotropic gelation of sodium alginate and pectin was used for the production of hydrogel particles from Immortelle (*Helichrysum italicum*) extract [128].

Becerril et al. [39] described in detail in their review emulsion techniques, core-shell nanofibers, cyclodextrins and liposomes, among others, as the most recent strategies used for encapsulating antimicrobial agents for their stable inclusion in packaging materials. The size and shape of the micro or nanoparticles produced were quite variable due to the encapsulating material and the microencapsulation method used for their preparations. The choice for the microencapsulation method is guided by cost, properties of the wall material and material to be encapsulated, the application and the desired application mechanisms.

Table 1 shows some examples of recent studies carried out with films made of agro-based polymers incorporated with encapsulated plant-derived bioactive compounds.

Once the bioactive compound is encapsulated, it can be incorporated into the film-forming solution directly [30,114,115,127] with later deposition of the resulting solution onto drying plates, or by sprinkling as recently reported by Nogueira et al. [29,117,118]. In the second method, the film-forming solution is initially deposited onto support plates and, then, the encapsulated bioactive compound is added by sprinkling through a stainless-steel sieve over the entire surface area of the film-forming solution already arranged on the plates. The authors studied this second method because they believed that, due to the hydrophilic characteristic presented by the blackberry pulp microparticles (water solubility of approximately 60%, [118]), there was a great possibility that they would dissolve when added and homogenized in the film-forming solution, which also had a hydrophilic characteristic. Taking that into consideration, in this second method, the stage that consisted of homogenizing the microparticles in the film-forming solution was eliminated, which allowed the microparticles to remain intact. Cross-sectional images of the films obtained by Scanning Electron Microscope revealed the presence of dispersed and/or agglomerated blackberry microparticles within the starch matrix when incorporated directly, and predominantly dispersed and/or agglomerated blackberry microparticles within the starch matrix when incorporated by sprinkling. Regardless of the incorporation method, it was observed that the microparticles remained mostly intact [29,117]. The location of the microparticles in the starch matrix was influenced by the method used for their incorporation into the film-forming solution, while the location of the microparticles in the polymeric matrix influenced the bioactive, barrier and mechanical properties.

## 5. Properties of Films from Agro-Based Polymers and Incorporated with Plant-Derived Bioactive Compounds

The final functionality of films is related to their bioactive properties (such as antioxidant, antimicrobial and antibrowning activities), their ability to serve as barrier to water vapor, oxygen, carbon dioxide and ultraviolet (UV–vis) light, water vapor permeability; mechanical properties (tensile stress, elongation at break) and other physical properties (such as opacity and color) [105]. The present review cites the properties most commonly evaluated in agro-based polymers films incorporated with plant-derived bioactive compounds.

### 5.1. Bioactive Properties

The use of plant-derived bioactive compounds with antioxidant and antimicrobial properties in biodegradable films as a sustainable strategy for maintaining and/or extending the shelf life of food products has been promising [28], since microbial growth and oxidation are largely responsible for the degradation of food products, which limit their conservation. One of the clear advantages of that use is to protect the packaged food without the addition of edible preservatives directly in its composition. This is because the incorporation of edible preservatives directly into the food has proved to have limited action due to the rapid diffusion of the bioactive substance from the surface to the mass of the product, leading to the concomitant loss of the effectiveness of its action [39].

The major concern regarding microbial growth, in addition to microbial deterioration (deteriorating microorganisms), which reduces the shelf life of various food products [130,131], is the presence of some microorganisms (pathogenic microorganisms) that can transmit diseases to consumers through food [132,133]. Oxidation, on the other hand, is generally considered the main cause of oil and fat rancidity, which is one of the most frequent mechanisms of food deterioration and reduced shelf life [134]. In addition to altering the taste (rancification) and nutritional quality (loss of essential vitamins and fatty acids) of foods, oxidation results in reactive and toxic compounds [135] that pose harm and become unsuitable for consumption.

Therefore, the use of natural antimicrobial and antioxidant compounds in biodegradable films tends to contribute to the preservation of food quality and safety. An example of this was observed when the incorporation of tea polyphenol into a film made with pomelo peel flour caused an antimicrobial effect on the film; as its concentration was increased from 5 to 20%, there was an increase in the inhibition zone for *Escherichia coli* and *Staphylococcus aureus* around the films. Films without tea polyphenol did not show antimicrobial effect, proving that it was the tea that generated this property [28].

In another study, films incorporated with essential oils from medicinal plants showed the ability to kill and inhibit the growth of Gram-positive and Gram-negative bacteria, with varying activity regarding the inhibition zone (from 18.50 to 38.83 mm) for each bacterium [3]. The bioactive compounds present in essential oils are capable of destroying bacterial cell integrity, making it permeable, in addition to inhibiting respiratory processes and ion transport, which may result in its death. In addition to presenting an antimicrobial effect, these films simultaneously presented antioxidant capacity [3].

The incorporation of blueberry extract into films containing isolated soy protein also transferred antioxidant capacity to the film, delaying the oxidation and hydrolysis of the pork fat packed with it during storage (36 °C, 40% relative humidity, for 5 weeks) [93]. Malherbi et al. [74] used films containing guabiroba pulp to store extra virgin olive oil for a period of 15 days. Although the guabiroba pulp is a rich source of bioactive antioxidant compounds, adding it to the films did not show any additional effect on the oxidative stability of this oil. Wu et al. [28], on the other hand, observed a significant decrease in the value of peroxides for soy oil packed with pomelo peel flours and tea polyphenol film stored in an oven at 50 °C for 30 days, showing that the bioactive compounds present in the film were able to delay oil oxidation during storage.

Films incorporated with mango, acerola (*Malpighia glabra* L.) or seriguela (*Spendias purpurea*) pulps caused an antioxidant effect on dendê oil during 40 days of storage and can be applied to control product oxidation. A reduction of 50%, 49% and 56% in the total phenolic compounds of the films incorporated with mango, acerola and seriguela pulps was observed during storage of dendê oil. This suggests the migration of these compounds from the films to the dendê oil during storage [94]; thus, the film can be considered active packaging. Other examples of most recent studies of plant-derived bioactive compounds incorporated into biodegradable films and their respective properties in active packaging are listed in Table 2.

**Figure 4:**
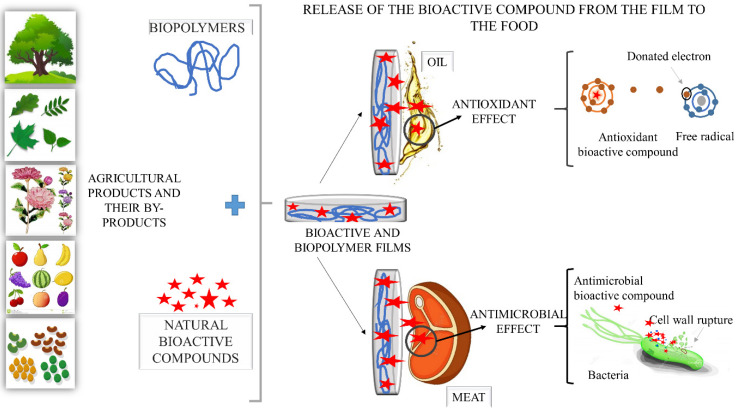
Illustration of the bioactive properties of films from agro-based polymers and incorporated with plant-derived bioactive compounds.

Active packaging, in addition to protecting food, as conventional packaging does, has acquired new functions, such as the release of antimicrobial and antioxidant agents onto food surfaces, bringing extra benefits to the packaged food product [138]. When bioactive films are applied in foods, their intentional interaction with the food (direct contact) and/or medium, empty packaging space (indirect contact) in which it is inserted leads to the release of bioactive compounds with different properties (antioxidant and or antimicrobial, among others) onto the food surface, where it will act to inhibit the growth of microorganisms and prevent oxidation of lipids, fats and other compounds [94,138], as shown in Figure 4. For this to happen, biopolymeric films must keep bioactive compounds (anthocyanins, flavonoids, vitamins, among others) bioavailable and in the best conditions until they are released into the food system. In addition, the release of bioactive compounds must be sufficiently abundant to guarantee their intended action, such as, for example, antioxidant or antimicrobial.

Thus, in order for the packaging system to achieve its intended purpose, knowledge of the controlled transfer mechanism of bioactive compounds to food is required, as well as of the microbial growth kinetics or lipid oxidation. When the rate of migration of an antimicrobial or antioxidant agent is faster than the growth rate of target microorganisms or lipid oxidation, the bioactive agent will be depleted before the expected storage period. As a result, the packaging system will lose its activity and this will cause the growth of microorganisms or lipid oxidation. On the other hand, when the release rate is very slow, microorganisms can grow before the antimicrobial agent is released, in the same way as lipid oxidation. Thus, the release rate of bioactive compounds from the film into the food must be specifically controlled to be similar to the growth rate of the target microorganisms and the oxidation rate of the target lipid compounds [102].

The mechanisms involved in the release of plant-derived bioactive compounds from films of agro-based polymers in direct contact with food or food simulats are: diffusion-induced release (bioactive compound diffuses through the micro or macro-porous structure of the polymer matrix and is transported away from the film surface into the food); swelling induced release (in this case, the bioactive compound has a low coefficient of diffusion so that its release occurs, the polymer matrix must be placed in a compatible liquid or wet food and it swells when fluid enters its matrix and, as result, the diffusion coefficient of the bioactive compound increases allowing it to diffuse out of the film); disintegration induced release (the main reasons for this type of release are the degradation, cleavage or deformation of polymer) [40]. The characteristics of bioactive compounds (molecular weight and volume, polarity, solubility, volatility, affinity) and film-forming material (pore size, polymeric chain flexibility, polarity and packing density) in food or food simulants (pH, water activity and temperature), along with the time of contact, will significantly influence the interactions between them, as well as the release of bioactive compounds to the food or food simulants [102], as demonstrated by several authors.

Piñeros-Hernandez et al. [81] studied the migration of rosemary polyphenols using water and 95% ethanol as simulants for aqueous and fatty foods, respectively. After 7 days of exposure to the simulants, it was observed that the films containing 4.4 and 13.6 mg (gallic acid equivalent—GAE/kg dried film) of polyphenols released 40 and 140 mg (gallic acid equivalent—GAE/kg water simulant) into the aqueous food simulant, respectively, whereas, only a small amount of polyphenols were detected in the fatty food simulant, less than 7 mg (gallic acid equivalent—GAE/kg 95% ethanol simulant) for both films. In ethanolic medium, a low amount of simulant penetrated the film matrix, unlike the aqueous medium, which quickly penetrated the matrix of cassava starch and glycerol, leading to the diffusion of a large amount of rosemary polyphenols into the simulant.

Kevij et al. [75] came across a different behavior; in their study, the release of curcumin from whey protein isolate films was faster and higher with the fatty food simulant (95% ethanol, fatty simulant medium) than with the semi-fatty food simulant (50% ethanol, semi-food simulant medium). This behavior is explained by the lipophilic nature and better solubility of curcumin in 95% ethanol [75]. It is known that, in addition to the chemical composition of the active compounds and the conditions of the surrounding environment [75,81], the polymer–active compound interactions and the structure of the film matrix also influence the release of active compounds from biopolymer films.

The curcumin released from the composite films into different food simulants is favored by lipophilic substances and can be controlled by reducing the polarity of the starch nanovehicle by acetylation [129]. The release of thymol from gelatin films was more influenced by the lecithin content used as an emulsifier than by the thymol content. Apparently, the highest level of lecithin (1%) decreased the gelatin network’s cohesiveness, which resulted in an increase in the film porosity, favoring the release of thymol [115]. The increase in the concentration of thymol also led to an increase in its diffusion to the film [115]. Mahcene et al. [3] observed that the amounts of essential oils from medicinal plants released from sodium alginate films increased when increasing the time of contact with water, reaching the maximum oil release rate at the hundredth minute, when the complete solubilization of the films occurred. The solubility of sodium alginate, a material used for the formation of films in water, seems to have contributed to this release. It is also possible that the volatility of oils also facilitated their release [3].

In general, the highest release rates of bioactive compounds to food or food simulants occur when the film-forming matrix is porous or soluble in the medium, or even when the bioactive compound has a high affinity with the surrounding medium. The longer the contact time between the film and the food or the food simulator, the greater the release rate of the compound. The strategy of using encapsulated bioactive compounds in films tends to generate greater protection from encapsulated compounds (degradation, volatilization, etc.) and a better-controlled release.

The encapsulation of marjoram essential oil with pickering emulsions provided a significantly slower release profile in pectin film samples in comparison to essential oil-loaded nanoemulsions [114]. Nieto-Suaza et al. [129] also observed that it was possible to control the release of curcumin-loaded starch nanoparticles incorporated into films with Aloe vera–banana starch in different food simulants, changing the characteristics of the incorporated nanovehicles. Films incorporated with curcumin-loaded native starch nanoparticles, after 168h, reached 38.2% and 57.1% of curcumin release into highly lipophilic food simulants (simulant 1, ethanol 50% v/v; simulant 2, oleic acid as vegetable oil), while only 32.2% and 47.2% were released from films containing curcumin-loaded acetylated starch nanoparticles. According to the authors, acetylated starch nanoparticles encapsulated higher values of curcumin than nanoparticles of native starch nanoparticles due to the reduction of the starch polarity by acetylation. Although the same amounts of nanoparticles were incorporated, films containing curcumin-loaded acetylated starch nanoparticles presented higher lipophilic level. This might have made it difficult to extract the curcumin molecules from the films to the simulants in comparison to the native starch nanoparticles that were more hydrophilic.

So, it is clear that the solubility of the encapsulating agent in the polymeric matrix and in the food simulants significantly influence the release rate of the bioactive compound from the film to the surrounding environment. This resource has great potential for controlling its release. In addition to the type of encapsulating agent used, the technique used for the incorporation of the encapsulated bioactive compounds into the film-forming solution also seems to influence its release rate.

Nogueira et al. [29,117] observed that films incorporated with microparticles by sprinkling had higher antioxidant capacity values than films with microparticles incorporated directly. The authors attributed this behavior to the fact that blackberry microparticles remained on the film surface, which may have facilitated the extraction of bioactive compounds due to the direct contact between the blackberry and the extraction solvents. As for the direct incorporation, the blackberry was integrated into the polymeric matrix. This probably hindered the extraction of bioactive compounds by the extraction solvents, since arrowroot starch may have functioned as a protective barrier [29,117].

The production of multilayer films has also been used as a strategy to modify the location of bioactive compounds in the polymer matrix and to improve the control of the release of bioactive compounds from the film to foods or food simulants. Layer-by-layer (LbL) deposition is one of the methods used in this production. The layers are formed one by one, being physically or chemically bonded together to form the final multilayer film [40,139]. Basically, the multilayer bioactive films produced are composed of three layers: a layer with high barrier properties used to prevent the loss of the bioactive compound to the environment; the matrix layer containing the bioactive compound, which has high diffusion; and a control layer that is in contact with the food and has less swelling capacity than the matrix layer, which allows the control of the release of the bioactive compound to the food surface [40,140]. Xia et al. [141] prepared edible multilayer films using zein and gelatin (outer layer of zein, intermediate layer of zein / gelatin and inner layer of gelatin) containing tea polyphenols in the middle and inner layer. Multilayer films exhibited a longer and slower release form compared to mono or bilayer films [141].

In addition to the location of bioactive compounds in the film-forming matrix, the way in which they are distributed also appears to affect the bioactivity of the film. Another major challenge is to be able to homogenously incorporate bioactive compounds into the film-forming biopolymer matrix. Studies have shown regions with particles of bioactive compounds agglomerated within the biopolymer matrices [26,90,117], or even segregated [115]. The lack of precision in the incorporation of bioactive compounds into the biopolymer generates variations in their distribution in the films, which contributes to the limitation in the standardization of their functional properties, as well as in the amounts of bioactive compounds released to packaged foods. It is known that the amount of any component that migrates to food depends on its initial concentration in the biopolymer and its solubility. Obviously, regions of film with particles of agglomerated or segregated bioactive compounds tend to have a higher concentration of bioactive compounds compared to other areas of the same film.

The more homogeneous the distribution of the bioactive compound in the film, the more efficient it will act on the food surface. For example, films containing allyl isocyanate (AIT) microemulsions showed stronger antimicrobial activity and were more homogeneous than those containing conventional emulsions. According to Guo [104], the fact that the particles are smaller allowed for a more homogeneous distribution of allyl isocyanate (AIT) encapsulated in microemulsions and micro-particles, and the presence of micro-pores with sizes ranging from 100 to 300 nm and micro-channels in the film matrix allowed the migration of the antimicrobial from the center to the film surface and its release into the liquid medium or food. This allowed the films to have a continuous antimicrobial effect on food during prolonged exposure.

Despite the promising results, more studies should be carried out to evaluate the relationship between the location and the distribution of bioactive compounds in the film with their release rate in food. The vast majority of works perform release tests using food simulants to understand the migration of bioactive compounds from films to different types of food. Migration is measured using chemical tests that are specific to quantify the bioactive compounds or group of bioactive compounds in question. This is because it is extremely difficult to measure the direct migration of bioactive compounds from a film to a food due to complex compositions (water, carbohydrates, fats, lipids, proteins, vitamins, fibers and minerals) found in most foods [138]. Generally, the release rate of bioactive compounds from the biopolymer film is evaluated by using food simulants, such as 95% ethanol and water, used as fatty and aqueous food simulants, respectively [81], under defined temperature, time and static or dynamic mode conditions. Nevertheless, the release characteristics of bioactive compounds using food simulants may not be totally faithful to those found when using real foods, due not only to the different compositions, but also to the variety of other intrinsic characteristics, such as density, viscosity, water content, pH, food temperature, among others.

In addition, these types of tests are especially difficult due to the large number of external variables that must be evaluated, such as time and temperature conditions, quantity, and space between the film and the food. All of these variables can lead to different types of results, which is why the standardization of the conditions adopted in the release tests is so necessary. Advances in the specificity, sensitivity and selectivity of the appropriate analytical techniques to determine the amount of bioactive compounds present in films and in food will allow quantification of their migration to real foods. These advances in analytical techniques will also enable the achievement of more accurate results. All of this will help to fill some of the gaps that still exist in searches, such as: the lack of knowledge on the ideal concentration of each bioactive compound for each type of food; the release kinetics of bioactive compounds for food products (liquid, semi-solid and solid); and mainly how to control the unintended migration of bioactive compounds to food.

### 5.2. Effect of Plant-Derived Bioactive Compounds on the Physical, Mechanical and Barrier Properties of Films from Agro-Based Polymers

The knowledge on the functional properties of films is of fundamental importance and scientific and technological interest due to the requirements that the different films must present to be used as food packaging, since many industrial applications depend on these properties. Among the main functions of films from agro-based polymers that will be used as food packaging are the mass transfer control (to avoid gain or loss of solutes and volatiles), mechanical protection (protecting the integrity of food and packaging), protection barrier (controlling the migration of water vapor and gases between the product and the environment), low water solubility (protecting from solubilization in water). The incorporation of plant-derived bioactive compounds in the film-forming solution, besides modifying the bioactive properties of resulting films, can concomitantly modify their structure and, as a result, modify their functionality, such as their ability to serve as a water vapor barrier; water vapor permeability; tensile strength; elongation at break; and their physical properties such as opacity, color and thickness [26,74].

Table 3 summarizes the effect of incorporating plant-derived bioactive compounds on the mechanical, barrier and physical properties of agro-based polymers films.

The increasing incorporation from 0 to 40% (mass/mass of biopolymer) of blackberry pulp into the arrowroot starch film-forming solution, apart from generating color with tones ranging from violet to magenta, typical of blackberry pulp, also promoted an increase in thickness (from 0.065 to 0.133 mm), elongation (from 3.18% to 13.59%), water vapor permeability (from 3.62 to 4.60 g.mm/m^2^·day·kPa), water solubility (from 14.18 to 25.46%), and reduced tensile strength (from 22.71 to 3.97 MPa) [26]. The increase in thickness and elongation at break, and the decrease in strength of films, produced with blends of gelatin and cornstarch were also observed with the addition of guabiroba pulp [74]. The increase in thickness, of films produced by the casting method, is generally related to the increase in solid content due to the addition of fruit pulp, extracts or plant-derived bioactive compounds in the same volume or mass of solution deposited on the support plate, as well as to the protuberances found on the film surface [26,103].

In general, bioactive compounds with hydrophilic characteristics tend to increase the water vapor permeability of the film, since the water vapor transfer is generally caused by the hydrophilic composition of the film and depends on the composition of hydrophilic to hydrophobic compounds in the film [105]. Furthermore, the increase in water solubility, water vapor permeability and elongation at break observed in films with fruit pulp and plant extracts may be consequences of the presence of structural defects in the polymer matrix, as well as on the surface of films, which make them less resistant to the passage of water molecules. For cassava starch films, the incorporation of polyphenols-rich rosemary extracts inhibited the bond between the molecules of glycerol and starch and led to the formation of cracks in these materials. As a result, the water vapor permeability and the mechanical properties of the active films were affected [81].

As for films made with fruit pulps, the discontinuity of the polymeric matrix may be due, not only to the incorporation of bioactive compounds, but also to other structures present in their composition, such as proteins, lipids, fibers, vitamins, minerals and sugars that fit the biopolymer matrix through different types of intermolecular interactions [26]. Another possible cause that has been reported is the plasticizing effect provided mainly by the sugars present in fruits when added to the biopolymer matrix [26,90,92].

**Figure 5:**
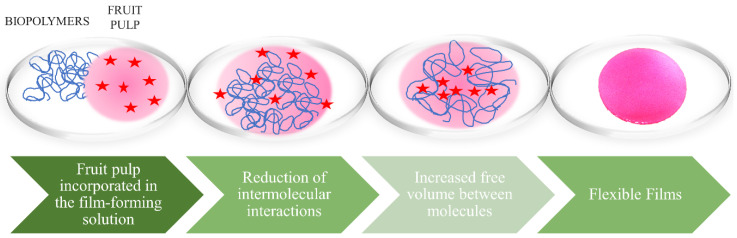
Plasticizing effect performed by the fruit pulp when incorporated into the biopolymer.

Plasticizers are used in films to improve both their physical and mechanical properties, joining possible cracks in the biopolymer matrix. When added to the film-forming solution, they modify the molecular organization of the network by increasing the free volume between the molecules. This action usually causes a reduction in intermolecular interactions between the adjacent chains of the biopolymer, increasing the mobility of these chains (Figure 5). Consequently, changes in the material occur, such as increased flexibility, extensibility and distensibility, followed by decreased mechanical strength, glass transition temperature, and gas and water vapor barriers [26,143].

Gamma-aminobutyric acid, a nutritive bioactive compound, in addition to generating antioxidant activity, also acted as a plasticizer when incorporated into the pectin film, proving to be an alternative to glycerol [123]. Hydroalcoholic residual extract obtained by cold pressing green coffee beans also showed a strong plasticizing effect when added into carboxymethyl cellulose films [31]. The use of mature coconut water (byproducts from coconut processing) as a solvent also reduced the amount of glycerol needed to form a satisfactory coconut protein film [78]. Thus, it is noted that the functional properties presented by the films are directly affected by the intermolecular interactions between the biopolymer and the bioactive compound.

In general, plasticizers increase flexibility and reduce the water vapor barrier. These are undesirable characteristics for films that will be applied to foods with intermediate humidity, which are characterized by high water activity (from 0.65 to 0.90) and an overall soft texture [105].

The incorporation of encapsulated plant-derived bioactive compounds into films made of agro-based polymers has also proved to be a viable alternative for improving the mechanical and barrier properties of films based on biodegradable biopolymers.

Wang et al. [37] reported that the addition of chitosan hydrochloride and chitosan carboxymethyl nanocomplexes containing anthocyanins, into gelatin films improved their mechanical properties, thermal stability and antioxidant capacity. The improvements in both the tensile strength and elongation at break of films were attributed to the homogeneous dispersion of nanocomplexes into the gelatin network and the interactions between the hydroxyl groups and the polar groups of gelatin. Chitosan films incorporated with a β-cyclodextrin inclusion complex of 2-phenyl ethanol were homogeneous, transparent and colorless, and showed high mechanical resistance [30]. The tensile strength increased from 34.5 to 48.8 MPa for chitosan films with the inclusion complex; Young’s modulus values also increased. The interactions between chitosan and the inclusion complex allowed the formation of a compact network, increasing film strength and stiffness [30].

The increasing incorporation of curcumin-loaded native and acetylated starch nanoparticles (0.01 to 0.1%) into films made with banana starch and Aloe Vera gel also increased their tensile strength from 3.74 to 5.01 MPa and 4.80 MPa, respectively. This improvement in tensile strength was attributed to the strong interaction between the native starch nanoparticles and the biopolymer matrix. The small size and the high presence of hydroxyl groups on the surfaces of native starch nanoparticles allowed the formation of many hydrogen bonds with the biopolymer matrix, resulting in an increase in tensile strength. Lower tensile strength values found in films with curcumin-loaded acetylated starch nanoparticles in comparison to native starch nanoparticles were attributed to fewer hydroxyl groups present on the surface of the nanoparticles, due to the starch acetylation process, which consequently generated a weaker interaction with the biopolymer matrix. Nevertheless, the increase in film stiffness, due to the incorporation of nanoparticles, reduced elasticity (elongation at break from 56.3 to 45.4%). The presence of highly hydrophobic curcumin molecules limited the interaction between water and the film matrix, leading to a decrease in water vapor permeability (from 4.59 to 2.12 × 10^9^ g·Pa^−1^·s^−1^·m^−1^) and water solubility (53.8% to 29.1%, at 95 °C). These results demonstrate that the properties presented by the films highly depend on the characteristics of the encapsulated bioactive compound, the characteristics of the encapsulating agent, and the compatibility between the encapsulating materials, bioactive compounds and the film-forming material [129].

Encapsulating some types of bioactive compounds has become essential to improve their compatibility with the film-forming polymer, which is the case of essential oils, for example. Their encapsulation improved their interaction with polymers of agricultural origin, which in their majority have hydrophilic characteristics. The addition of microencapsulated oregano essential oil also improved the mechanical properties and reduced the water vapor permeability of films. Although the microparticles are large (250 ± 6 μm), the good interaction between soy protein concentrate, a material used for forming the film, and the alginate used as a wall material in the microencapsulation of the oil resulted in a more continuous film matrix with few cavities, which led to improvements to them [127]. The incorporation of gelatin microparticles, containing papaya skin as a filling material, into films also made from gelatin allowed a good interaction between these materials, resulting in a more continuous film matrix and an increase in tensile strength and Young’s modulus [144].

For Almasi et al. [114], the application of Pickering emulsions with marjoram essential oil into films made with pectin produced a highly dense and less permeable structure that resulted in good mechanical and water barrier properties for the film produced. The compatibility between pectin and loaded nanocarriers was confirmed by the Fourier transform infrared spectroscopy, X-ray diffraction and field emission scanning electron microscopy.

Li et al. [115] observed that the incorporation of thymol nanoemulsions (co-emulsified by blend of gelatin and soy lecithin) weakened the hydrogen bonds of the gelatin molecules and decreased the cohesiveness of the biopolymer network, causing an increase in the water vapor permeability and elongation at break of gelatin film and a decrease in the moisture content and tensile strength. Nevertheless, due to the fact that the particles are uniform and nano-scale, smooth and continuous surfaces were observed for gelatin films containing thymol nanoemulsions [115]. On the other hand, the incorporation of α-tocopherol nanocapsules into carboxymethyl cellulose films led to a decrease in water vapor permeability, tensile strength and Young’s modulus. The incorporation of nanocapsules caused porosity and changes in the film matrix structure [116]. Porous and heterogeneous surface with the presence of hydrophobic masses was observed for quinoa and chitosan films added with thymol nanoemulsions, suggesting that oil droplets aggregated during the film drying process [38].

The location of the particles of bioactive compounds in the films, within the polymeric matrix or on its surface, also influence the properties presented by them. Films incorporated directly into encapsulated blackberry pulp were less soluble in water than films incorporated with blackberry powder by spraying and the location of these film particles influenced this behavior. The fact that blackberry dust particles remained on the film surface when incorporated by spraying allowed its direct contact with water leading to solubilization, given its porous and hydrophilic characteristic, which consequently generated holes on the film; as a result, the water molecules entered the starch matrix and led to its solubilization. Water vapor permeability was also affected. The increase in blackberry particles in the film led to their aggregation. The aggregation of particles reduces the interaction between the active surface area and the polymeric matrix, decreasing its cohesiveness and consequently increasing water vapor permeability [117].

## 6. Limitations to be Overcome for Future Advances

Agro-based polymers incorporated with plant-derived bioactive compounds have great advantages in relation to synthetic plastic, as they are environmentally friendly and have characteristics of edibility, biocompatibility and natural bioactive properties. However, despite their many advantages, their use as bioactive food packaging on an industrial scale is still limited by several factors. The main factors that limit its commercial success are listed and described in this section.

With the exception of starch, raw materials of agricultural origin, such as cellulose, alginate sodium, pectin, chitosan, gums and proteins, such as whey, soy, gluten and gelatin, among others, the isolation process of these polymers can require high energy consumption and expensive chemicals, which affect the economic and environmental values of the films produced with them. The great variability regarding the physical–chemical composition of the origin of both the agricultural polymers and the plant-derived bioactive compounds also hinder the standardization of the bioactive functional properties, water vapor permeability, tensile strength, elongation at break, as well as of the physical properties of films produced with them. The current laboratory-scale production of agro-based films incorporated with plant-derived bioactive compounds has problems such as the inability to make continuous films, long drying times and imprecise thickness control. Thus, to overcome these issues, better and cheaper production methods are needed along with investments in scaling machines so that the films produced can compete with the current ones, not only in terms of efficiency, but also in terms of price [73].

In addition, as agro-based polymers are mostly hydrophilic, films produced with them have lower properties than plastics derived from petroleum, especially in terms of mechanical strength, such as insufficient stretching, and poor water vapor barrier properties [73].

These properties can still be impaired by the heterogeneous dispersion [26,90,117] of bioactive compounds in the film-forming matrix, or by the incompatibility between them [38], and the potential losses of bioactive compounds, due to their volatilization, degradation by heat and light, when incorporated into the film-forming solution [30,39], must be overcome in order to guarantee the production of films with bioactive properties. Although encapsulation of the bioactive compound improves its stability, it is important that the methods and encapsulating agents applied are effective and inexpensive, so that these films can compete with current ones [39]. However, more research is needed to determine the ideal concentration of each bioactive compound for each type of food so that the antimicrobial or antioxidant action is detected, the release profile of the bioactive compounds from the film to the food is understood, along with how to guarantee a controlled release (time x quantity) during the entire storage period and distribution time of the food to the consumer [39,102]. Therefore, a good understanding of all these factors is necessary for films produced with agro-based polymers incorporated with plant-derived bioactive compounds to be effectively used as active food packaging on a commercial level.

## 7. Conclusions

Agro-based polymers, i.e., starch, sodium alginate, pectin, chitosan, cellulose, whey protein, gelatin, soy and gluten proteins have been extensively used for the production of environment-friendly food films or packaging. In addition, the combination of agro-based polymers and derived-plant bioactive compounds (phenolic compounds, carotenoids, vitamins, among others) allows the development of bioactive films with antioxidants, antimicrobial activity and innovative colors. Different methods have been used for the production of bioactive films, such as the use of inherently bioactive biopolymer materials, the direct incorporation of free or encapsulated bioactive compounds into the film-forming solution and the spraying of free or encapsulated bioactive compounds onto the film-forming solution already on the support plates, among others. The functional, barrier and resistance properties presented by the films are directly influenced by the interactions between the bioactive compounds and the film-forming solution. In general, films produced with agro-based polymers still have limited mechanical and barrier properties when compared to synthetic plastic films. The use of bioactive films to increase the shelf life, stability and safety of foods has proved to be promising. In addition, the strategic use of bioactive films as vehicles and targeted delivery of bioactive compounds to food has proved to be useful for the stability and safety of foods that have been packaged with them. The improved stability and controlled release rate provided by the films allow the bioactive compounds to be transferred from the film to the surface of the food, where they start to act, preventing the growth of microorganisms and the oxidation of compounds, such as lipids. However, information on the mechanisms involved in this process is still limited especially regarding the controlled release of the bioactive compound into the food. Although films have several positive characteristics such as biodegradability and bioactive properties, they are still produced on a laboratory scale, and issues related to processing difficulties, high cost, and difficulties in standardizing film properties need to be resolved so that their production can move onto an industrial scale.

## Figures and Tables

**Table 1:** Recent examples of films with encapsulated plant-derived bioactive compounds from plant-derived developed by their incorporation into the agro-based polymer during production process.

Encapsulated Bioactive Compound	Encapsulation Method	Film-Forming Biopolymer	Film Production Method	Incorporation of Bioactive Compound Method	Reference
2-phenyl ethanol is a natural compound, which has many applications due to its nice fragrance, bacteriostatic and antifungal character.	β-Cyclodextrin inclusion complex of bioactive compounds, followed by spray drying	Chitosan	Solution casting	directly into the film-forming solution	[30]
Marjoram essential oil and Pickering emulsions	Marjoram essential oil-loaded nanoemulsions (NE) and Pickering emulsions (PE). Low molecular surfactant of Tween 80 and blend of whey protein isolate (WPI) and inulin were used for preparation of NE and PE, respectively.	Citrus pectin	Solution casting	directly into the film-forming solution	[114]
Thymol essential oil antimicrobial	Thymol nanoemulsions co-emulsified by natural emulsifiers (i.e., a blend of gelatin and soy lecithin)	Gelatin (type B)	Solution casting	directly into the film-forming solution	[115]
Vitamin E (α-tocopherol)	Nanoencapsulation	Carboxymethyl cellulose	Solution casting	directly into the film-forming solution	[116]
Oregano essential oil	Microencapsulated by ionic gelation	Soy protein concentrate	Casting technique	directly into the film-forming solution	[127]
Blackberry pulp	Microencapsulated by freeze drying and spray drying	Arrowroot starch	Casting technique	directly and by sprinkling into the film-forming solution	[29]
Curcumin, known for its antimicrobial and antioxidant activity	Native green banana starch and acetylated banana starch nanoparticles were synthetized by nanoprecipitation	Banana starch and *Aloe vera* gel	Casting technique	directly into the film-forming solution	[129]
Immortelle (*Helichrysum italicum*) extract	The preparation of hydrogel particles was carried out by using internal ionotropic gelation of sodium alginate and pectin.	Sodium alginate and Low metoxyl pectin	Casting method	directly into the film-forming solution	[128]
Cranberry extract as a source of anthocyanins	Nanocomplexes of chitosan hydrochloride, carboxymethyl chitosan and anthocyanins were fabricated through electrostatic interaction	Bovine gelatin with 250 Bloom value	Casting method	directly into the film-forming solution	[37]
Phenolic compounds of fresh rice grass were extracted	The microencapsulation consisting of extract solution and maltodextrin was prepared using the freeze-drying method	Carboxymethyl cellulose blended	Casting method	directly in the film-forming solution	[126]
Thymol	Thymol nanoemulsions were produced by spontaneous emulsification, ultrasound, and a combination of both methods	Quinoa protein/chitosan	Casting method	directly into the film-forming solution	[38]

**Table 2:** Selected examples of bioactive compounds from plant-derived incorporated into biodegradable films and their respective properties in active packaging.

Source Material	Bioactive Compounds	Film-Forming Material	Effect on Food Packaging	Target Microorganism	Reference
Turmeric rhizome	Curcumin	Whey protein isolate	Antioxidant properties	-	[75]
*R. officinalis* L. *A. herba alba Asso* *O. basilicum* L. *M. pulegium* L.	1,8-cineole, alpha-thujone, linalool, pulegone (compounds in greater quantities)	Sodium alginate	Antibacterial effect and antioxidant capacity	6 bacterial: *Staphylococcus aureus*, *Escherichia coli, Salmonella entérica*, *Enterococcus faecium*, *Klebsiella pneumoniae* and *Enterococcus faecalis*	[3]
*Acca sellowiana* waste by-product (feijoa peel flour)	Phenolic e flavonoids (polyphenolic compounds)	Blends of pinhão starch citric pectin	Antimicrobial and Antioxidant properties	*Escherichia coli, Salmonella typhimurium* and *Pseudomonas aeruginosa*.	[80]
Extract of germinated fenugreek seeds	Phenolic compounds	Semi-refined κ-carrageenan	Antioxidant capacity Antimicrobial	Total bacterial count on chicken breast samples packed in active films	[107]
Green coffee oil	Fatty acids and phenolic compounds	Carboxymethyl cellulose	Antioxidant capacity	-	[31]
Guabiroba pulp	Phenolic compounds and ascorbic acid	Blends of corn starch and gelatin	Antioxidant effect	-	[74]
Blackberry pulp	Anthocyanins (phenolic compound)	Arrowroot starch	Antioxidant capacity	-	[26]
Tea	Polyphenol	Pomelo peel flours	Antioxidant activity and antimicrobial activity	*Escherichia coli* *Staphylococcus aureus*	[28]
Seaweeds (*Himanthalia elongata* and *Palmaria palmata*) and seaweed extracts	Phenolic compounds	Chitosan	Antioxidant capacity		[136]
Coconut water concentrate	Phenolic compounds	Coconut protein	Antioxidant capacity	-	[78]
Babassu mesocarp flour and starch	Phenolic compounds	Babassu mesocarp flour and starch	Antioxidant activity	-	[35]
Apple	Polyphenols	Chitosan	Antioxidant and antimicrobial properties	*Escherichia coli*, *Listeria monocytogenes* *Staphylococcus aureus,* *Colletotrichum fructicola*, *Botryosphaerial dothidea* and *Alternaria tenuissima*	[106]
Spirulina extract	Phenolic compounds	Crab chitosan	Antioxidant activity	*Escherichia coli*, *Staphylococcus aureus*, *Pseudomonas aeruginosa*, *Listeria monocytogenes*, *Salmonella typhimurium*, *Bacillus subtilis* and *Bacillus cereus*.	[137]

**Table 3:** Recent publications on the effect of incorporating plant-derived bioactive compounds on the mechanical, barrier and physical properties of agro-based polymers films.

Film-Forming Material	Plant-Derived Bioactive Compounds	Film Production Method	Incorporation of Bioactive Compound Method	Obtained Results	Reference
Turmeric flour produced from the turmeric dye extraction residue	Turmeric	Casting	Producing the film using inherently bioactive biopolymeric materials from agricultural by-products	The pH and temperature (T) used in the solubilization of the flour affected the functional and mechanical properties of the films. When T of 85.1 °C and pH of 8.1 were used, the films produced showed high mechanical resistance (9 MPa), low solubility (37%) and low permeability to water vapor (0.352 g mm h^−1^ m^−2^ kPa^−1^).	[34]
Babassu mesocarp flour or with starch isolated from babassu mesocarp	Babassu	Casting	Producing the film using inherently bioactive biopolymeric materials from agricultural by-products	Babassu flour films were less mechanically resistant, more flexible, more opaque, more hydrophilic, more permeable to water vapor and with greater water absorption capacity than babassu starch films.	[35]
Red rice flour and starch	Red rice (*Oryza glaberrima*)	Casting	Producing the film using inherently bioactive biopolymeric materials from agricultural products	Film thickness ranged from 0.242 mm to 0.297 mm. Red rice flour films were more permeable to water vapor (1.56 g mm h^−1^ m^−2^ kPa^−1^), soluble in water (24.59%) compared to the starch films (permeable to water vapor of 1.25 g mm h^−1^ m^−2^ kPa^−1^, solubility in water of 21.05%). The tensile strength (TS), young’s modulus (YM) and elongation at break (EAB) values of the rice starch film were higher than the values found for the rice flour film.	[113]
Whey protein isolate	Curcumin	Casting	Directly in the film-forming solution	The addition of curcumin into the film-forming formulations decreased the average thickness, water vapor permeability, and elasticity of the films, whereas the redness, yellowness, and strength considerably increased.	[75]
Sodium alginate	Essential oils of *R. officinalis* L., *A. herba alba Asso*, *O. basilicum* L. and *M. pulegium* L.	Casting	Directly into the film-forming solution	The essential oils were uniformly dispersed in the polymeric matrix and slightly improved the barrier properties, while decreasing the tensile strength of the film of sodium alginate.	[3]
High methoxyl pectin from citrus peels	Gamma-aminobutyric acid (GABA)	Casting	Directly into the film-forming solution	The increasing incorporation from 0 to 15% of GABA in the pectin film promoted an increase in elongation (from 8.78 to 32.15%), and reduced its tensile strength (from 6.41 to 1.43 MPa), water vapor permeability (from 3.59 to 2.55 × 10^−10^g m^−1^ s^−1^ Pa^−1^) water solubility (from 92.30 to 59.85 %).	[123]
Arrowroot starch	blackberry pulp	Casting	Directly into the film-forming solution	Increasing the concentration of blackberry pulp (from 0 to 40%, mass/mass of dry starch) in the film resulted in increased thickness (from 0.065 to 0.133 mm), increased elongation (from 3.18 to 13.59%), decreased tensile strength (from 22.71 to 3.97 MPa), increased water vapor permeability (from 3.62 to 4.60 g.mm/m^2^.day.kPa) and solubility in water (from 14.18 to 25.46%).	[26]
Corn starch and chitosan	Turmeric	Casting	Directly into the film-forming solution	High percentage of water absorption obtained for the Chitosan/starch films, despite the addition of turmeric (Chitosan/starch: 1841 ± 52% and Chitosan/starch/turmeric: 1939 ± 478%).	[32]
Blends of pinhão starch citric pectin	*Acca sellowiana* waste by-product (feijoa peel flour)	Casting	Directly in the film-forming solution	The incorporation of feijoa (0, 0.4, 1, 2, 3 and 4%, *w*/*w*) waste by-product increased the thickness of the films of 0.097 to 0.183 mm and decreased solubility in water of 6.00 to 4.24%. Films with 2% (665.00 ± 36.77 MPa) and 3% of feijoa (571.00 ± 29.70 MPa) presented the highest Young’s modulus.	[80]
Chitosan	Young apple polyphenols	Casting	Directly into the film-forming solution	The addition of Young apple polyphenols (0.25%, 0.50%, 0.75%, and 1.0% (*w*/*v*)) resulted in a significant increase in the thickness, density, swelling degree, solubility and opacity of chitosan film, but the water content, water vapor permeability and mechanical properties of the film were decreased.	[106]
Blend of pomelo peel flour and sodium alginate	Tea polyphenol	Casting	Directly into the film-forming solution	Addition of 10% tea polyphenol led to stronger intermolecular interactions and more compact microstructure, and thus enhanced the water barrier properties and tensile strength of the films.	[28]
Semi-refined κ-carrageenan	Water extract of germinated fenugreek seeds	Casting	Directly into the film-forming solution	Films incorporated with different concentrations of water extract of germinated fenugreek seeds were stand-alone films and had good flexibility, smoothness, homogeneous texture, and were easily removed from the casting plates.	[107]
Spider crab (*Maja crispata*) chitosan	*Spirulina* extract	Casting	Directly into the film-forming solution	The incorporation of *Spirulina* extract (from 0 to 20, (*w*/*v*) decreased water vapor permeability of 0.5242 to 0.3786 g mm d^−1^ Pa^−1^ m^−2^, and increased the tensile strength of 21.24 to 29.65 MPa and elongation at break of 26.13 to 34.20% chitosan films.	[137]
Citrus pectin	Nanoemulsion and Pickering emulsion stabilized marjoram (*Origanum majorana* L.) essential oil	Casting	Directly into the film-forming solution	The thickness value of film samples showed a significantly increasing trend by incorporation of Pickering emulsion stabilized marjoram oil. Addition of nanoemulsion and Pickering emulsion stabilized marjoram essential oil and decreased water vapor permeability of 9.96 × 10^−7^ g/m.h.Pa (Control film) by 4.27 × 10^−7^ g/m.h.Pa and 1.19 × 10^−7^ g/m.h.Pa, respectively.	[114]
Sodium alginate and Low metoxyl pectin	Immortelle (*Helichrysum italicum*) extract	Casting	Directly in the film-forming solution	The incorporation of hydrogel particles, whose average size was 1940 μm (alginate particles) and 3080 μm (pectin particles), increased the thickness of the developed films.	[128]
Gelatin	Thymol nanoemulsions	Casting	Directly into the film-forming solution	The incorporation of thymol nanoemulsions increased the thickness (from 0.076 for 0.103 mm), water vapor permeability (from 8.83 to 10.09 g/(m·s·Pa) × 10^−11^) and elongation at break (from 46.05 to 111.02%) of gelatin film, but decreased the moisture content (from 16.46 to 11.14 g/100g) and tensile strength (from 9.77 to 2.19 MPa).	[115]
Carboxymethyl cellulose	Nanoencapsulated vitamin E (α-tocopherol)	Casting	Directly into the film-forming solution	The addition of α-tocopherol nanocapsules decreased the water vapor permeability (from 0.223 to 0.114 × 10^−7^ g m m^−2^ s^−1^ Pa^−1^) and tensile strength (from 37.076 to 23.105 MPa), while elongation at break increased from 32.30 to 52.97%.	[116]
Starch-carboxy methyl cellulose	Rosemary essential oils encapsulated in chitosan nanogel	Casting	Directly into the film-forming solution	The films incorporated with encapsulated rosemary essential oils had higher solubility in water and water vapor permeability in comparison to the oil-free films.	[142]
Aloe vera/banana starch	Curcumin-loaded native and acetylated starch nanoparticles	Casting	Directly into the film-forming solution	The increase in the concentration of nanoparticles generated an increase in tensile strength from 3.74 MPa in films without nanoparticles to 5.01 MPa and 4.80 MPa at the highest concentration of Curcumin-loaded native and acetylated starch nanoparticles, respectively.	[129]
Soy protein concentrate	Free and encapsulated oregano essential oil	Casting	Directly into the film-forming solution	The addition of free oregano essential oil reduced the tensile strength and increased the water vapor permeability of the Soy protein concentrate film, whereas the microencapsulated oil increased the tensile strength and decreased the water vapor permeability.	[127]
Gelatin	Anthocyanins nanocomplexes	Casting	Directly into the film-forming solution	The addition of anthocyanin nanocomplexes increased the tensile strength and elongation at break of the gelatin films	[37]
Chitosan	2-phenyl ethanol and β-Cyclodextrin inclusion complex of 2-phenyl ethanol	Casting	Directly in the film-forming solution	The addition of 2-phenyl ethanol did not change tensile strength or elongation at break of the chitosan film. Tensile strength value increased from 34.5 to 48.8 MPa for the chitosan films with the inclusion complex, but without glycerol.	[30]
Arrowroot starch	Microencapsulated blackberry pulp by spray drying	Casting	Incorporated directly and by sprinkling into the film-forming solution	Films incorporated with blackberry particles showed an increase in thickness, water solubility, water vapor permeability and elongation at break, as well as a decrease in tensile strength in comparison to the control film. Films with blackberry powder (30 and 40% of blackberry solids mass/biopolymer mass) incorporated by sprinkling had the highest elongation results in comparison to the other films.	[118]
Arrowroot starch	Free and microencapsulated blackberry pulp powders by freeze-drying	Casting	Incorporated directly and by sprinkling in the film-forming solution	The addition of free and microencapsulated blackberry pulp increased the thickness and water solubility and decreased tensile strength of arrowroot starch film. Films added with blackberry powder by sprinkling were more soluble in water and permeable to water vapor than films incorporated with blackberry directly.	[117]
